# Viral vector-based imaging uncovers infection-induced re-localization of pepino mosaic virus proteins

**DOI:** 10.1128/jvi.01694-25

**Published:** 2026-03-24

**Authors:** Jing Zhang, Zhaolei Li, Hao He, Xueping Zhou, Fangfang Li

**Affiliations:** 1 State Key Laboratory for Biology of Plant Diseases and Insect Pests, Chinese Academy of Agricultural Sciences, Institute of Plant Protection 12661, , Beijing, China; 2 State Key Laboratory of Rice Biology, Zhejiang University, Institute of Biotechnology 12377, Hangzhou, Zhejiang, China; Tsinghua University, Beijing, China

**Keywords:** pepino mosaic virus (PepMV), viral vector, dynamic subcellular localization, movement protein 2 (TGB2), viral replication

## Abstract

Defining the subcellular localization of viral proteins is crucial for understanding viral pathogenesis. Pepino mosaic virus (PepMV), a significant pathogen of solanaceous crops, remains poorly characterized in terms of its protein localization and potential as a viral vector, limiting our understanding of its pathogenesis. Transient expression systems often fail to fully recapitulate PepMV infection dynamics. To address this, we developed recombinant viral vectors using the virus’s own promoters, enabling a direct comparison of protein localization between transient expression and viral infection. Here, we found that viral infection dramatically altered the localization of movement proteins (TGB1, TGB2, TGB3), inducing perinuclear aggregates associated with viral replication and increasing their association with peripheral puncta. In contrast, the distribution of RNA-dependent RNA polymerase (RdRP) and coat protein (CP) remained relatively stable. Of note, a key technical achievement was creating a PepMV-based vector (PepMV^B^) for stable TGB2 expression. By adding a subgenomic promoter downstream of the CP open reading frame to drive green fluorescent protein (sGFP)-tagged TGB2, we achieved, for the first time, stable visualization of TGB2 during systemic infection, showing its incorporation into large, stable perinuclear structures associated with viral replication. In summary, this study provides an optimized PepMV-based vector system that better captures viral protein dynamics during infection, overcoming limitations of transient expression. This platform is a valuable tool for investigating viral replication and movement, offering insights for antiviral strategy development.

**IMPORTANCE:**

Pepino mosaic virus (PepMV) is a major economic pathogen causing significant losses in global greenhouse tomato production. A major challenge in understanding PepMV pathogenesis has been the lack of experimental systems that accurately capture the dynamic behavior of viral proteins within living plants during infection. Conventional transient expression approaches often incompletely reflect infection-associated protein dynamics. Here, we develop a PepMV-based viral vector that, for the first time, enables visualization of key viral protein localization during active PepMV infection, including stable imaging of the movement protein TGB2 during systemic spread. Using this system, we provide new insights into the spatial organization of viral replication- and movement-associated proteins within host cells. This optimized platform offers a robust and physiologically relevant platform for future mechanistic studies and may facilitate the identification of novel antiviral strategies for tomato crops.

## INTRODUCTION

Pepino mosaic virus (PepMV), a positive-sense single-stranded RNA virus belonging to the genus *Potexvirus* (family *Alphaflexiviridae*), was first identified in pepino (*Solanum muricatum*) in 1980 (1). It has since emerged as a major pathogen of greenhouse tomato production worldwide, causing significant economic losses (2, 3). PepMV infects a broad range of solanaceous hosts, including tomato (*Solanum lycopersicum*), potato (*Solanum tuberosum*), and pepper (*Capsicum annuum*) (2, 4). Furthermore, its increasing detection in mixed infections with viruses like Southern tomato virus and tomato brown rugose fruit virus often exacerbates symptom severity and worsens economic losses (5, 6).

The PepMV genome is approximately 6.4 kb in length and encodes five major open reading frames (ORFs) (7). The 5′-proximal ORF encodes the RNA-dependent RNA polymerase (RdRP), which features methyltransferase (MET), helicase (HEL), and polymerase (POL) domains. The RdRP is essential for viral replication, catalyzing the synthesis of both genomic and subgenomic RNAs (8). Notably, ectopic expression of the POL domain can induce cell death (9). Downstream, three overlapping ORFs constitute the triple gene block (TGB), encoding proteins TGB1, TGB2, and TGB3, that collectively facilitate viral cell-to-cell movement and replication complex assembly (10). TGB1 is a multifunctional protein possessing nucleic acid-binding activity and RNA silencing suppression capacity (10, 11). Furthermore, TGB3 has been identified as a key symptom determinant, with a single amino acid at position 67 critical for inducing necrotic symptoms (12). The 3′-terminal ORF encodes the coat protein (CP). Beyond its canonical role in packaging the viral genome (13), the CP is indispensable for both local and systemic movement and functions as an RNA silencing suppressor (14, 15).

Current understanding of the subcellular localization of PepMV proteins remains limited, with existing studies providing only partial insights. Available evidence shows that TGB1 localizes to both the cytoplasm and the nucleus, where it can form inclusion bodies reminiscent of viral replication or movement complexes (15). In addition, CP has been reported to associate with viral replication sites, and physical interactions between PepMV TGB1 and CP have been demonstrated *in planta* (15, 16). In contrast, more detailed mechanistic insights have been obtained from the related potexvirus potato virus X (PVX). PVX TGB1 induces the formation of “X-bodies” through membrane remodeling and mediates extensive reorganization of the host actin cytoskeleton and endomembrane system, thereby facilitating the assembly of viral inclusion bodies and intracellular trafficking (17, 18). TGB2 and TGB3 associate with the endoplasmic reticulum (ER), promote the assembly of viral replication complexes, and guide TGB1 to plasmodesmata (19). In PVX, TGB2 primarily forms irregular perinuclear aggregates, with a smaller fraction distributed as cytoplasmic granules, and precisely delineates the surface of viral replication compartments containing RdRP and dsRNA. These structures are subsequently enveloped by TGB3 (20). However, whether analogous mechanisms occur in PepMV infection represents an important area for further investigation.

Plant virology has advanced considerably in recent years, with research directions becoming increasingly diverse (21). In this context, basic studies on plant viruses are also gaining renewed attention (22). Plant viruses are also monitored by intracellular immune surveillance systems mediated by nucleotide-binding leucine-rich repeat (NLR) receptors, providing a broader biological context for studies of viral protein behavior during infection (23).

To address this gap, we employed a two-step experimental strategy. First, we used transient expression, both with and without PepMV co-infection, to examine the initial localization and accumulation patterns of individual viral proteins. Second, we engineered infectious PepMV clones carrying fluorescent protein tags, enabling live-cell imaging of replication- and movement-associated proteins under physiologically relevant infection conditions. This approach provides a dynamic and biologically meaningful framework for functional studies of PepMV proteins and supports the development of targeted antiviral strategies. Notably, the TGB2-specific viral vector developed in this study offers a powerful tool for characterizing this key movement protein in its native context. Moreover, the same vector engineering strategy is broadly applicable to functional analyses of TGB modules in other virus genera, such as *Carlavirus* and *Hordeivirus*.

## RESULTS

### Subcellular localization of PepMV proteins in transient expression assays

Subcellular localization provides critical insights into viral protein function. To examine the localization of PepMV-encoded proteins in the absence of viral infection, we expressed N- and C-terminal yellow fluorescent protein (YFP) fusions in RFP-H2B transgenic *Nicotiana benthamiana* leaves via *Agrobacterium tumefaciens*-mediated transient expression (24). Fluorescent signals were observed using confocal laser scanning microscopy at 48 and 72 h post-infiltration (hpi). These transient expression assays provide a baseline reference for subcellular distribution in the absence of viral replication and coordinated intracellular trafficking.

**Fig 1:**
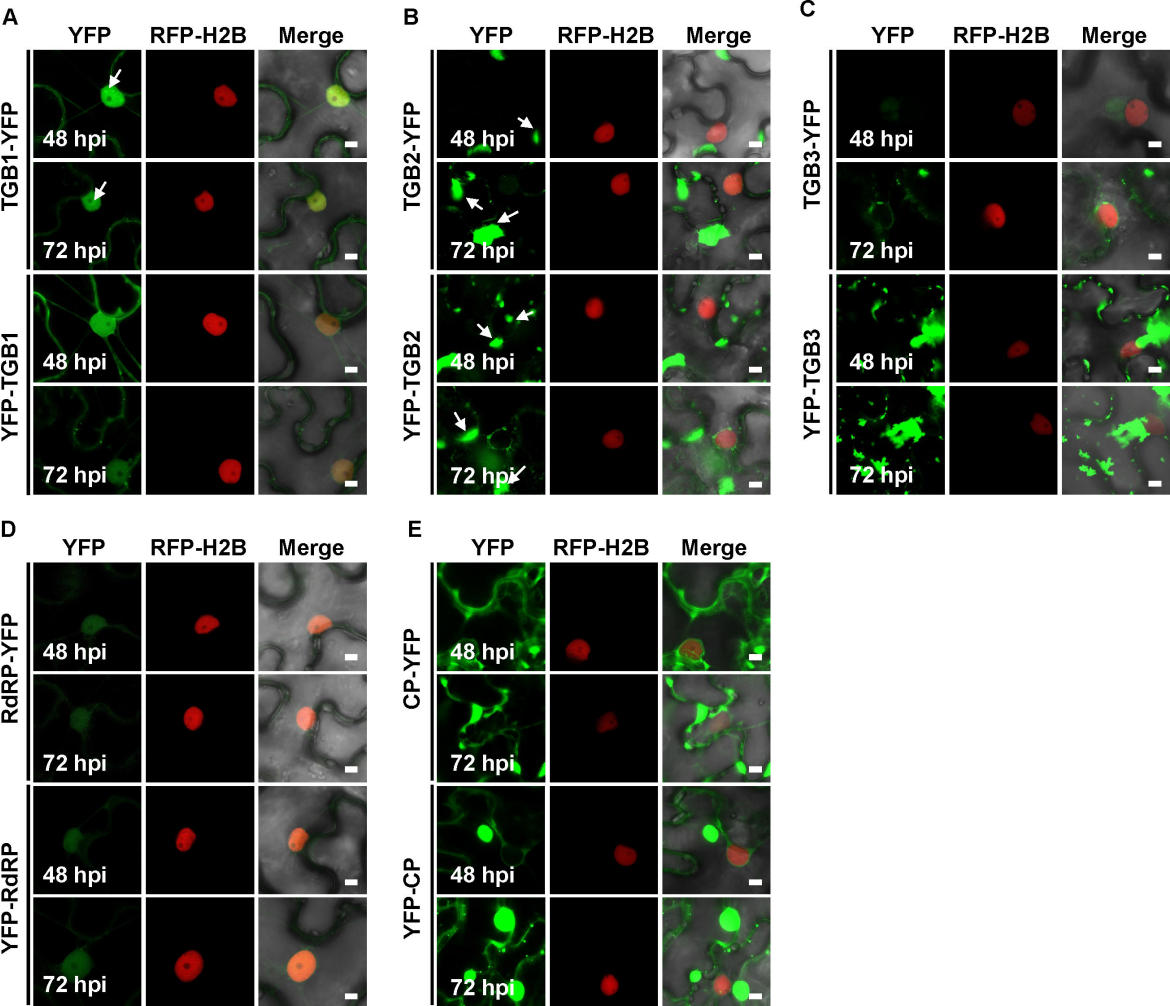
Subcellular localization of yellow fluorescent protein (YFP)-fused PepMV proteins in *N. benthamiana* leaf epidermal cells. *A. tumefaciens* cultures expressing the indicated YFP-fusion proteins (TGB1, TGB2, TGB3, RdRP, or CP) were infiltrated into leaves of *N. benthamiana* plants stably expressing the nuclear marker RFP-H2B. (**A**) TGB1-YFP and YFP-TGB1. (**B**) TGB2-YFP and YFP-TGB2. (**C**) TGB3-YFP and YFP-TGB3. (**D**) RdRP-YFP and YFP-RdRP. (**E**) CP-YFP and YFP-CP. Confocal laser scanning microscopy images were captured at 48 and 72 hpi. YFP signal is shown in green, and nuclei are marked by RFP-H2B (red). White arrows in panels **A** and **B** indicate representative punctate aggregates in the nucleus and cytoplasm, respectively. All images within a panel were acquired and processed under identical settings. Scale bar = 5 µm (applies to all panels). Images are representative of three independent biological replicates (*n* > 20 cells per replicate).

TGB1 fusion proteins localized to both the nucleus and cytoplasm at 48 and 72 hpi, with nuclear distribution influenced by the YFP tag position. Both TGB1-YFP and YFP-TGB1 exhibited continuous fluorescence signals along the cell periphery and formed filamentous structures extending between the nuclear envelope and the plasma membrane. Within the nucleus, TGB1-YFP showed diffuse fluorescence with superimposed puncta (indicated by white arrows in Fig. 1A), whereas YFP-TGB1 was uniformly distributed without aggregation (Fig. 1A).

TGB2 fusion proteins were localized exclusively to the cytoplasm at 48 hpi, forming irregular aggregates. YFP-TGB2 also displayed punctate signals along the cell periphery. By 72 hpi, TGB2-YFP produced punctate signals on the nuclear periphery, with representative examples indicated by white arrows in Fig. 1B, while maintaining irregular cytoplasmic aggregates and acquiring peripheral puncta. YFP-TGB2 also exhibited nuclear periphery puncta under these conditions, and both fusions were associated with reticular structures (Fig. 1B).

In contrast, TGB3 localization was strongly influenced by the position of the YFP tag. At 48 hpi, TGB3-YFP formed one or two irregular aggregates in the cytoplasm, whereas YFP-TGB3 produced numerous cytoplasmic aggregates and exhibited abundant punctate signals at the cell periphery. By 72 hpi, both fusions localized to the nuclear envelope and were associated with reticular structures. Both also showed distinct punctate signals along the cell periphery, though YFP-TGB3 exhibited more abundant peripheral puncta than TGB3-YFP (Fig. 1C).

Consistent with previous reports (25), both RdRP-YFP and YFP-RdRP localized predominantly to the nucleus, with weak cytoplasmic signals at 48 and 72 hpi (Fig. 1D). CP localization was markedly influenced by YFP tag position. CP-YFP showed minimal temporal changes, exhibiting continuous linear and punctate signals along the cell periphery, cytoplasmic aggregates associated with reticular structures, and consistent nuclear envelope localization at both time points. In contrast, YFP-CP displayed all localization features observed for CP-YFP, but additionally formed regular spherical aggregates. By 72 hpi, YFP-CP showed enhanced peripheral punctate signals and a significant increase in spherical aggregates compared to 48 hpi (Fig. 1E).

### PepMV infection alters the subcellular localization and accumulation of viral proteins

To systematically assess how viral infection influences the subcellular localization and accumulation dynamics of PepMV-encoded proteins, we performed *A. tumefaciens*-mediated co-expression assays in transgenic RFP-H2B *N. benthamiana* leaves. *A. tumefaciens* cultures expressing the YFP-fused viral proteins were co-infiltrated with PepMV infectious clones, and subcellular localization was examined by confocal laser scanning microscopy. Parallel western blot analysis of leaf extracts at multiple time points enabled quantitative assessment of protein accumulation. To specifically evaluate the effect of viral replication, YFP-fusion proteins were expressed with or without PepMV co-infection, followed by immunoblotting.

**Fig 2:**
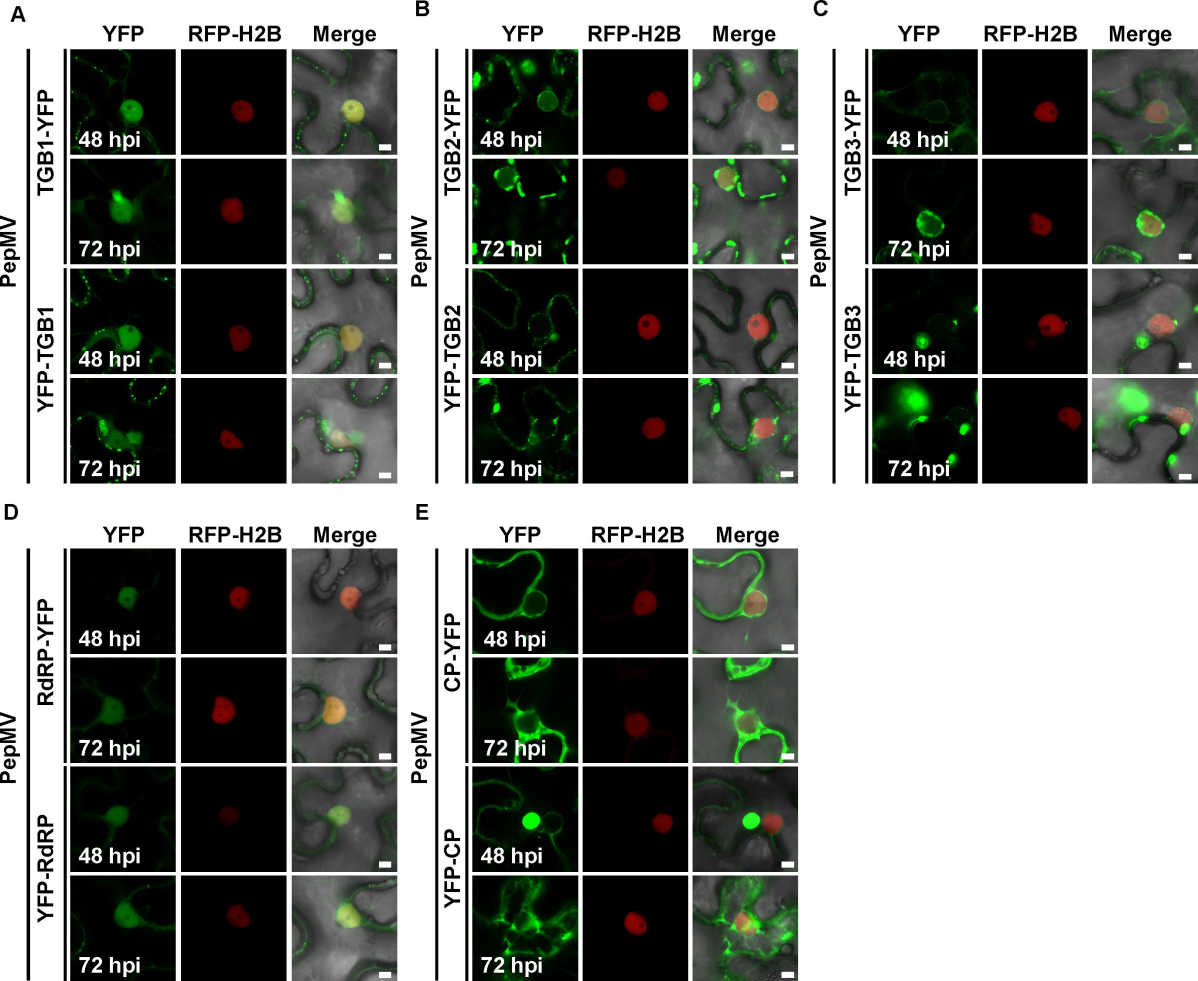
Subcellular localization of PepMV viral proteins fused to YFP in the presence of viral infection. *A. tumefaciens* cultures carrying constructs for the expression of the indicated YFP-fusion proteins were infiltrated into leaves of *N. benthamiana* plants stably expressing the nuclear marker RFP-H2B. Simultaneously, *A. tumefaciens* carrying a PepMV infectious clone (OD₆₀₀ = 0.5) was co-infiltrated. (**A**) TGB1-YFP and YFP-TGB1. (**B**) TGB2-YFP and YFP-TGB2. (**C**) TGB3-YFP and YFP-TGB3. (**D**) RdRP-YFP and YFP-RdRP. (**E**) CP-YFP and YFP-CP. All Agrobacterium suspensions for protein expression were adjusted to an OD₆₀₀ of 0.5, and 0.5 mL of each culture was infiltrated per leaf sample. Confocal microscopy images were captured at 48 and 72 hpi. The YFP signal is shown in green, and nuclei are marked by RFP-H2B (red). All images within a panel were acquired and processed under identical settings. Scale bar = 5 µm (applies to all panels). Images are representative of three independent biological replicates (*n* > 20 cells per replicate).

Viral infection markedly altered TGB1 localization. Although both TGB1-YFP and YFP-TGB1 maintained nuclear and cytoplasmic distribution during infection, their patterns diverged substantially from those in virus-free conditions. Compared to expression without PepMV (Fig. 1A), TGB1-YFP co-expressed with PepMV intensified punctate signals at the cell periphery by 48 and 72 hpi and formed prominent perinuclear aggregates by 72 hpi (Fig. 2A). Similarly, YFP-TGB1, which showed no punctate signals along the cell periphery without virus at 48 hpi (Fig. 1A), developed abundant signals under infection, with perinuclear aggregates also evident by 72 hpi.

TGB2 localization was also substantially modified in the presence of the virus. In contrast to the diffuse cytoplasmic aggregation observed without PepMV (Fig. 1B), TGB2-YFP co-expressed with the virus localized to the nucleus, perinuclear region, and cell periphery, displaying distinct punctate signals as early as 48 hpi (Fig. 2B). YFP-TGB2 similarly exhibited punctate signals at both perinuclear and in the cell periphery, with nuclear envelope association, detected earlier during infection. Although perinuclear aggregates at 72 hpi were less prominent than those of TGB2, they represented a clear increase in size and fluorescence intensity compared to the 48 hpi time point (Fig. 2B).

The subcellular localization of TGB3-YFP was significantly altered in the presence of PepMV. At 48 hpi, it localized to the nuclear envelope, exhibited continuous signal along the cell periphery, and was associated with reticular structures (Fig. 2C). This contrasted with the virus-free condition, where it formed only a few cytoplasmic aggregates and showed no prominent nuclear envelope association (Fig. 1C). By 72 hpi, viral infection induced the formation of distinct perinuclear aggregates (Fig. 2C). YFP-TGB3 also developed perinuclear aggregates under infection conditions at 72 hpi but lost the nuclear peripheral localization observed in the absence of virus (Fig. 1C and 2C).

In contrast, RdRP fusion proteins maintained predominant nuclear localization with weak cytoplasmic signals at both time points during infection, showing no notable difference from virus-free conditions (Fig. 1D and 2D). CP fusion proteins also exhibited minimal localization changes, maintaining similar patterns at the cell periphery, nuclear envelope, and in cytoplasmic aggregates regardless of viral co-expression (Fig. 1E and 2E). A notable exception was the appearance of perinuclear CP aggregates at 72 hpi, which were not observed without virus (Fig. 1E and 2E).

In the presence of PepMV, all viral fusion proteins except RdRP displayed time-dependent accumulation, with protein levels increasing significantly from 48 to 72 hpi (Fig. S1F through J). In contrast, when expressed alone, accumulation profiles varied. TGB1 and TGB2 declined progressively between 48 and 72 hpi (Fig. S1A and B), while TGB3 and CP increased over the same period (Fig. S1C and E). Notably, YFP-RdRP and RdRP-YFP were only detectable in the presence of PepMV at 48 hpi (Fig. S1D).

### Subcellular localization of TGB1 and TGB3 in PepMV vector-based expression

**Fig 3:**
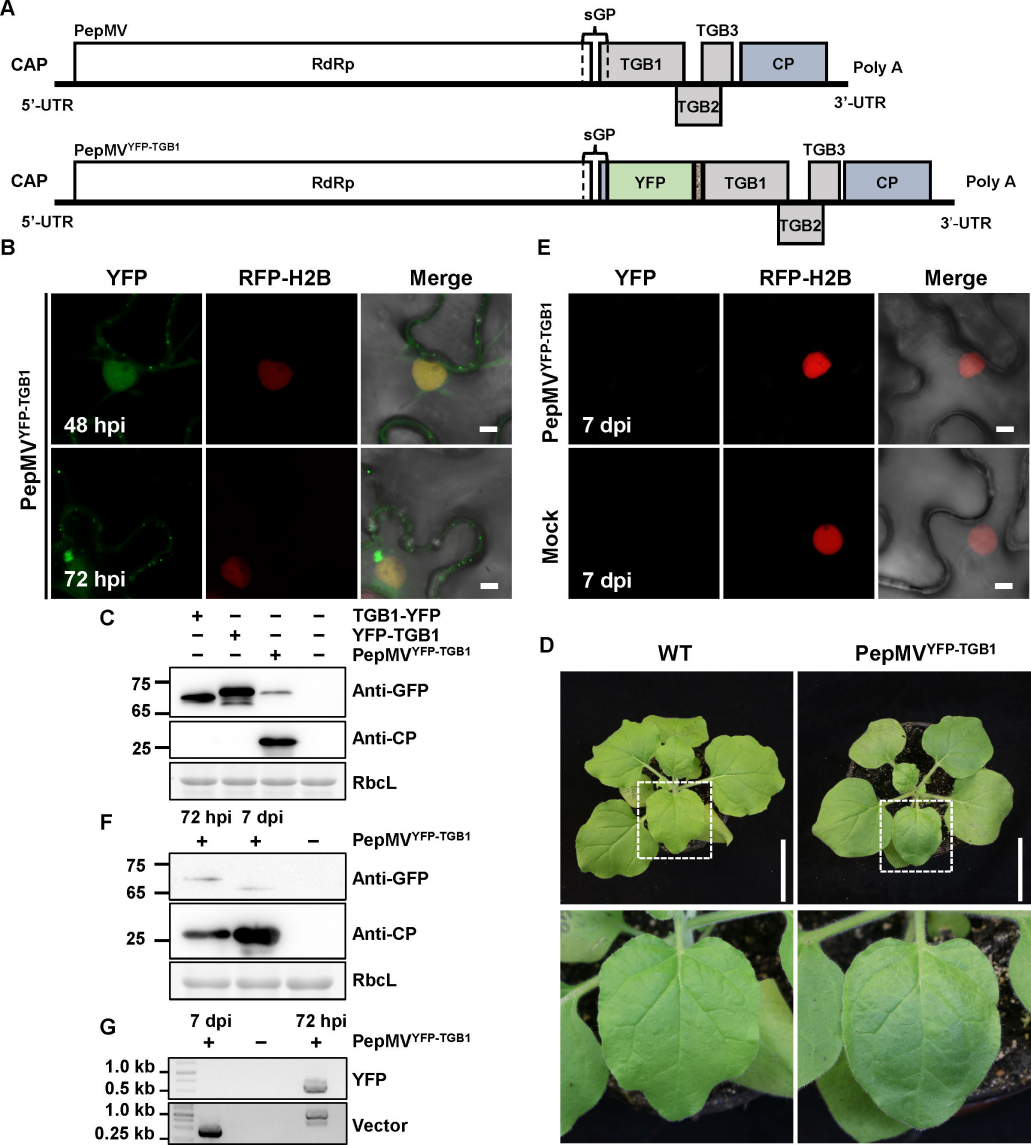
Analysis of PepMV-based vectors expressing TGB1-fused YFP. (**A**) Schematic representation of the wild-type PepMV genome (6.4 kb) and the recombinant PepMV^YFP-TGB1^ vector, drawn to scale. The YFP open reading frame was fused to the *TGB1* gene via a flexible 3Gly linker. (**B**) Confocal laser scanning microscopy images of RFP-H2B transgenic *N. benthamiana* leaves following agroinfiltration with the recombinant PepMV^YFP-TGB1^ vector or control constructs. Images were captured at 48 and 72 hpi. Scale bar = 5 µm. (**C**) Immunoblot analysis of fusion protein expression in infiltrated leaf tissues at 72 hpi using an anti-GFP antibody. (**D**) Representative symptoms of systemically infected leaves at 7 dpi. Scale bar = 6 cm. (**E**) Immunoblot analysis of protein expression in systemically infected leaves (7 dpi) using anti-GFP and anti-CP antibodies. (**F**) Confocal laser scanning microscopy images of systemic leaves at 7 dpi. Scale bar = 5 µm. (**G**) RT-PCR detection of viral RNA and verification of insert integrity. Total RNA from systemic leaves (7 dpi) was analyzed by RT-PCR with primer sets specific for the PepMV vector backbone and the YFP insert. All *A. tumefaciens* suspensions for infiltration were adjusted to an OD₆₀₀ of 0.5. A volume of 0.5 mL of each *A. tumefaciens* suspension was infiltrated per leaf. Experiments were repeated three times independently. For microscopy, more than 20 cells were examined per sample per replicate; representative images are shown.

**Fig 4:**
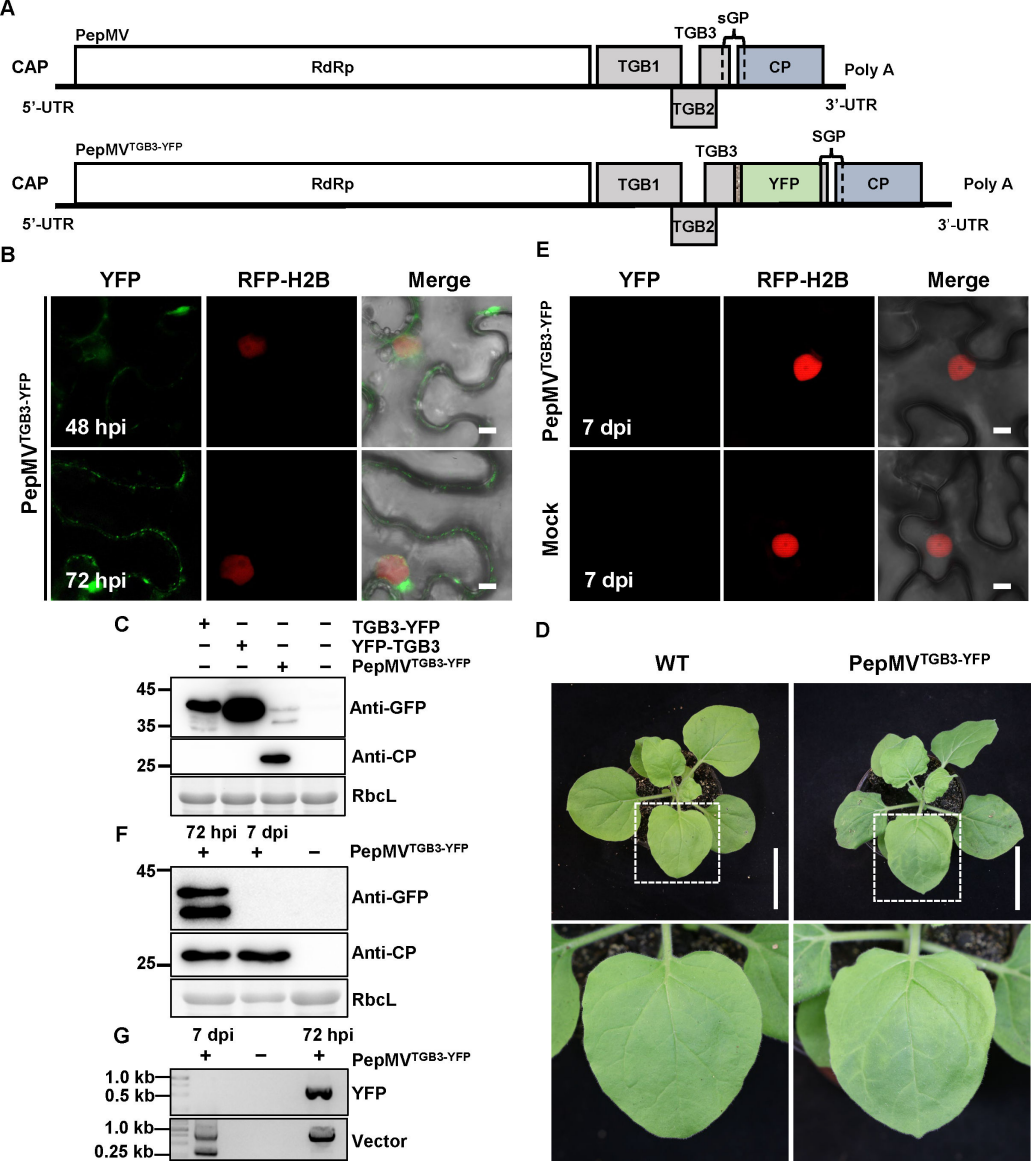
Analysis of PepMV-based vectors expressing TGB3-fused YFP. (**A**) Schematic representation of the wild-type PepMV genome (6.4 kb) and the recombinant PepMV^TGB3-YFP^ vector. The YFP open reading frame was fused to the *TGB3* gene via a flexible 3Gly linker. (**B**) Confocal laser scanning microscopy images of RFP-H2B transgenic *N. benthamiana* leaves following agroinfiltration with the recombinant PepMV^TGB3-YFP^ vector or control constructs. Images were captured at 48 and 72 hpi. Scale bar = 5 µm. (**C**) Immunoblot analysis of fusion protein expression in infiltrated leaf tissues at 72 hpi using an anti-GFP antibody. (**D**) Representative symptoms of systemically infected leaves at 7 dpi. Scale bar = 6 cm. (**E**) Confocal laser scanning microscopy images of systemic leaves at 7 dpi. Scale bar = 5 µm. (**F**) Immunoblot analysis of protein expression in systemically infected leaves. (**G**) RT-PCR detection of viral RNA and verification of insert integrity. Total RNA from systemic leaves (7 dpi) was analyzed by RT-PCR with primer sets specific for the PepMV vector backbone and the YFP insert. All *A. tumefaciens* suspensions for infiltration were adjusted to an OD₆₀₀ of 0.5. A volume of 0.5 mL of each bacterial suspension was infiltrated per leaf. Experiments were repeated three times independently. For microscopy, more than 20 cells were examined per sample per replicate; representative images are shown.

To investigate the native subcellular dynamics of TGB1 and TGB3 during PepMV infection, we engineered PepMV-based viral vectors harboring YFP fusions. These fusions were delivered via *A. tumefaciens*-mediated infiltration into RFP-H2B transgenic *N. benthamiana* plants. First, we generated a PepMV viral vector (PepMV^YFP-TGB1^; Fig. 3A) designed to express an N-terminal YFP fusion to TGB1, with the two domains connected via a flexible 3xglycine (3Gly) linker, from the native TGB1 subgenomic promoter. The fusion protein exhibited nucleocytoplasmic localization with persistent punctate signals at the plasma membrane throughout the observation period. By 72 hpi, irregular aggregates formed along the nuclear periphery (Fig. 3B). This spatial distribution was largely consistent with that observed during transient co-expression of YFP-TGB1 with PepMV (Fig. 2A), although protein accumulation levels in the viral vector system were significantly reduced (Fig. 3C). The reduced protein accumulation under viral expression conditions might better reflect the physiological levels encountered during actual viral infection. For TGB3, a C-terminal fusion to YFP, connected via a 3Gly linker (PepMV^TGB3-YFP^; Fig. 4A), the subcellular localization of the viral vector-driven TGB3 fusion was markedly different from that observed upon transient co-expression of TGB3-YFP with PepMV. In sharp contrast to the abundant cytoplasmic aggregates formed by transiently expressed TGB3-YFP under viral infection conditions, the PepMV^TGB3-YFP^ displayed a predominant perinuclear distribution with only a few cytoplasmic aggregates, which became conspicuous only by 72 hpi (Fig. 4B). Furthermore, the viral vector system revealed enhanced targeting to plasmodesmata and a reduced diffuse cytoplasmic signal. This more defined subcellular localization occurred despite markedly lower overall accumulation of the fusion protein from the viral vector compared to the transient expression system, suggesting that the observed pattern represents a more physiologically relevant state. By 72 hpi, both PepMV^YFP-TGB1^ and PepMV^TGB3-YFP^ formed prominent perinuclear aggregates resembling viral replication complexes (VRCs) (Fig. 3B and 4B). Both fusions supported systemic infection, inducing typical mosaic symptoms in upper leaves by 7 days post-infiltration (dpi) (Fig. 3D and 4D). However, neither fluorescent signals nor fusion proteins were detected in these leaves by confocal laser scanning microscopy or immunoblotting (Fig. 3E and F; 4E and F). RT-PCR assays confirmed the presence of viral genomic RNA but not the *YFP* insert (Fig. 3G and 4G), suggesting that the *YFP* tag was lost during systemic infection.

### Engineering a PepMV-based vector system for TGB2 localization studies

**Fig 5:**
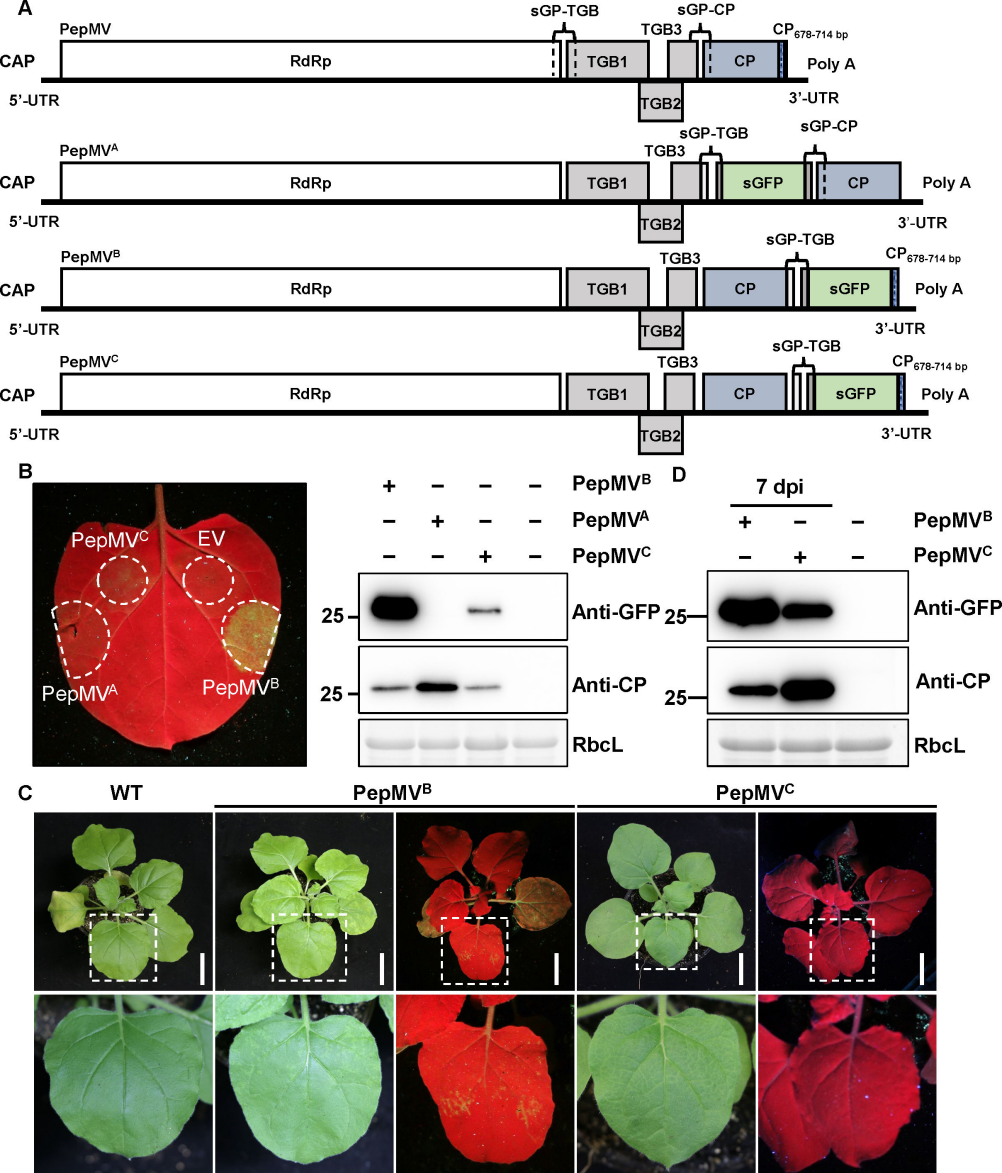
Analysis of PepMV-based vectors expressing sGFP. (**A**) Schematic representation of the wild-type PepMV genome and the modified vectors PepMV^A^, PepMV^B^, and PepMV^C^. The sGFP open reading frame, driven by a subgenomic promoter, was inserted downstream of TGB3 (PepMV^A^), downstream of CP (PepMV^B^), and following the 3′ UTR (PepMV^C^). (**B**) Transient expression analysis in *N. benthamiana* leaves. Leaves were infiltrated with *A. tumefaciens* cultures carrying the indicated constructs. At 3 dpi, sGFP expression in infiltrated leaves was visualized under UV light with a yellow filter (left). The empty vector pGR (Mock, EV) served as a negative control. Protein extracts from these leaves were analyzed by immunoblotting using anti-GFP antibodies to detect sGFP fusion proteins (right panel). (**C**) Representative images of *N. benthamiana* leaves infiltrated with *Agrobacterium* cultures carrying PepMV^B^ or PepMV^C^, visualized under UV light at 3 dpi. Scale bar = 6 cm. (**D**) Immunoblot analysis of systemic leaf protein extracts. Total proteins were extracted from systemically infected leaves and analyzed using anti-GFP antibodies to detect the expression and stability of the sGFP fusion proteins. All *A. tumefaciens* suspensions were adjusted to an OD₆₀₀ of 0.5 before infiltration. Immunoblotting assays were performed with three independent biological replicates, with each sample derived from three individually infiltrated leaves. Representative images are shown.

**Fig 6:**
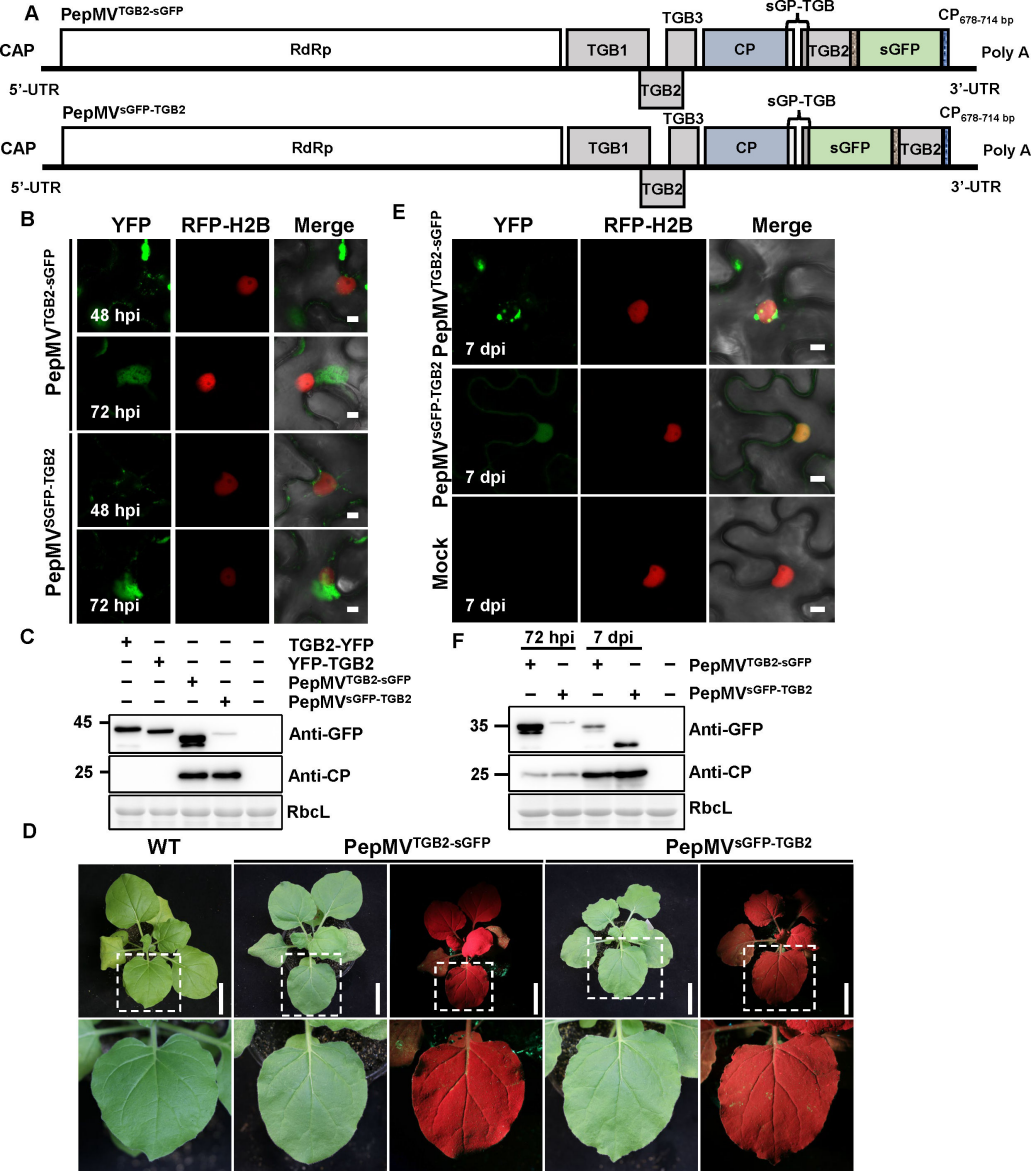
Analysis of PepMV-based vectors expressing TGB2-fused sGFP. (**A**) Schematic representation of the wild-type PepMV genome and the modified vectors PepMV^TGB2-sGFP^ and PepMV^sGFP-TGB2^. The sGFP open reading frame from tobacco mosaic virus (TMV)-GFP was fused to the *TGB2* gene via a flexible 3Gly linker in both configurations. (**B**) Subcellular localization of fusion proteins. RFP-H2B transgenic *N. benthamiana* leaves were infiltrated with *A. tumefaciens* suspensions carrying the indicated constructs and examined by confocal laser scanning microscopy at 48 and 72 hpi. Scale bar = 5 µm. (**C**) Immunoblot analysis of fusion protein expression in infiltrated leaf tissues at 72 hpi. (**D**) Representative symptoms of systemically infected leaves at 7 dpi. Scale bar = 6 cm. (**E**) Confocal laser scanning microscopy analysis of systemic leaves at 7 dpi. RFP-H2B transgenic *N. benthamiana* leaves were infiltrated with *A. tumefaciens* cultures, and systemic leaves were examined at 7 dpi. Scale bar = 5 µm. (**F**) Immunoblot analysis of protein expression in systemically infected leaves at 7 dpi. All *A. tumefaciens* suspensions were adjusted to an OD₆₀₀ of 0.5. A volume of 0.5 mL of each *A. tumefaciens* suspension was infiltrated per leaf. Experiments were repeated three times independently. For microscopy, more than 20 cells were examined per sample per replicate; representative images are shown.

Direct fluorescent tagging of TGB2 at its termini is not feasible due to its overlapping coding sequences with TGB1 and TGB3 (Fig. 6A), as this would interfere with the expression of these adjacent genes. To circumvent this limitation, we developed an internal insertion strategy by integrating a green fluorescent protein (sGFP) expression cassette driven by the native subgenomic promoter controlling TGB gene expression into three distinct sites of the PepMV genome: downstream of TGB3 (PepMV^A^), downstream of CP (PepMV^B^), and following the 3′ UTR (PepMV^C^) (Fig. 5A). Following *A. tumefaciens*-mediated infiltration of *N. benthamiana* leaves, UV imaging at 72 hpi revealed strong green fluorescence in PepMV^B^-infiltrated tissues, weak fluorescence in PepMV^C^, and no detectable signal in PepMV^A^ (Fig. 5B). Immunoblot analysis corroborated these observations (Fig. 5B). Both PepMV^B^ and PepMV^C^ systemically infected plants and accumulated sGFP fusion proteins in upper leaves; however, only PepMV^B^ exhibited sustained and intense fluorescence (Fig. 5C and D). Owing to its optimal balance between infectivity and transgene expression stability, PepMV^B^ was selected as the platform for subsequent TGB2 tagging studies. This platform, therefore, provides a functional viral background suitable for visualizing TGB2 dynamics during active PepMV infection.

Having established PepMV^B^ as a stable expression platform during systemic infection (Fig. 5), we next applied it to address the specific challenge of visualizing TGB2. The overlapping coding sequences of TGB1, TGB2, and TGB3 preclude direct terminal fluorescent tagging in the native viral genome. Using this optimized PepMV^B^ expression system, we engineered two distinct TGB2 fusion constructs. For an N-terminal fusion (PepMV^sGFP-TGB2^; Fig. 6A), a cassette containing the native TGB subgenomic promoter driving the expression of sGFP fused to TGB2 via a 3Gly linker was inserted downstream of the CP ORF. For a C-terminal fusion (PepMV^TGB2-sGFP^; Fig. 6A), the TGB2 ORF was fused directly to sGFP via a 3Gly linker; this fusion was expressed from the native promoter elements governing its original genomic context. Their subcellular localization closely matched the patterns observed under transient expression with PepMV infection (Fig. 2B). At 48 hpi, both fusions displayed perinuclear and irregular cytoplasmic aggregates. By 72 hpi, PepMV-driven fusions formed larger and more stable perinuclear aggregates resembling VRCs (Fig. 6B), in sharp contrast to the more diffuse patterns in transient assays. Immunoblotting confirmed the presence of full-length sGFP-fused proteins for both fusions, confirming intact expression at these time points (Fig. 6C). Notably, although both PepMV^TGB2-sGFP^ and PepMV^sGFP-TGB2^ supported systemic infection at 7 dpi (Fig. 6D), only PepMV^TGB2-sGFP^ maintained stable fluorescence, proper subcellular localization, and expected size of TGB-sGFP protein in systemically infected leaves, as validated by confocal laser scanning microscopy and immunoblotting (Fig. 6E and F). In contrast, PepMV^sGFP-TGB2^ exhibited aberrant nuclear localization in systemic leaves (Fig. 6E), and immunoblot analysis detected truncated sGFP products (Fig. 6F).

### Subcellular localization of RdRP and CP in PepMV vector-based expression

**Fig 7:**
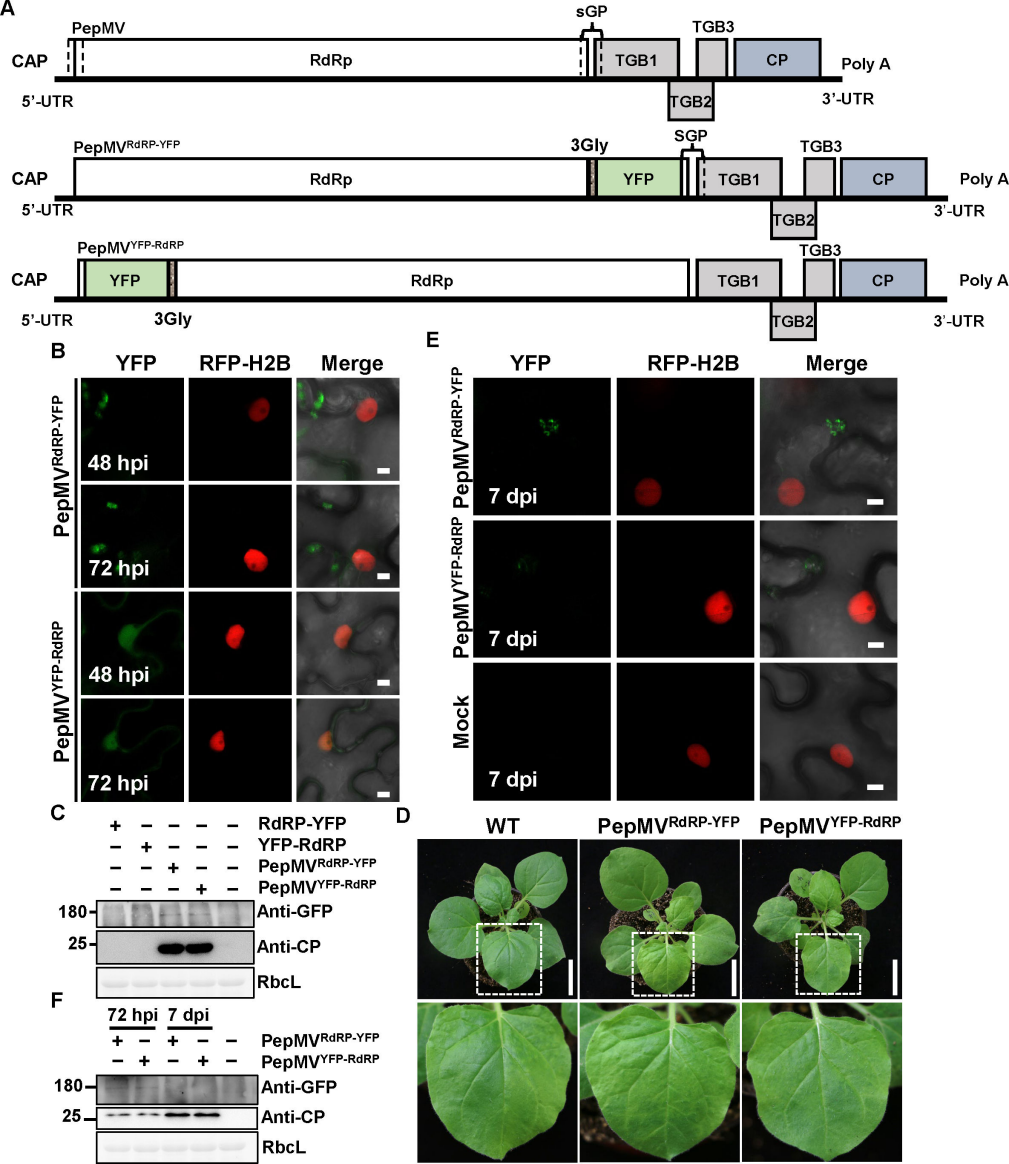
Analysis of PepMV-based vectors expressing RdRP-fused YFP. (**A**) Schematic representation of the wild-type PepMV genome and the modified vectors PepMV^RdRP-YFP^ and PepMV^YFP-RdRP^. The first dashed box in the wild-type genome represents the cis-acting elements required for RNA replication. The YFP open reading frame was fused to the *RdRP* gene via a flexible 3Gly linker in both configurations. (**B**) Confocal laser scanning microscopy images of RFP-H2B transgenic *N. benthamiana* leaves following infiltration with *A. tumefaciens* suspensions carrying the indicated constructs. Images were captured at 48 and 72 hpi. Scale bar = 5 µm. (**C**) Immunoblot analysis of fusion protein expression in infiltrated leaf tissues at 72 hpi using an anti-GFP antibody. (**D**) Representative symptoms of systemically infected leaves at 7 dpi. RFP-H2B transgenic *N. benthamiana* leaves were infiltrated and photographed. Scale bar = 6 cm. (**E**) Confocal laser scanning microscopy analysis of systemic leaves at 7 dpi. Scale bar = 5 µm. (**F**) Immunoblot analysis of protein expression in systemically infected leaves at 7 dpi. All *A. tumefaciens* suspensions were adjusted to an OD₆₀₀ of 0.5. A volume of 0.5 mL of each suspension was infiltrated per leaf. Experiments were repeated three times independently. For microscopy, more than 20 cells were examined per sample per replicate; representative images are shown.

**Fig 8:**
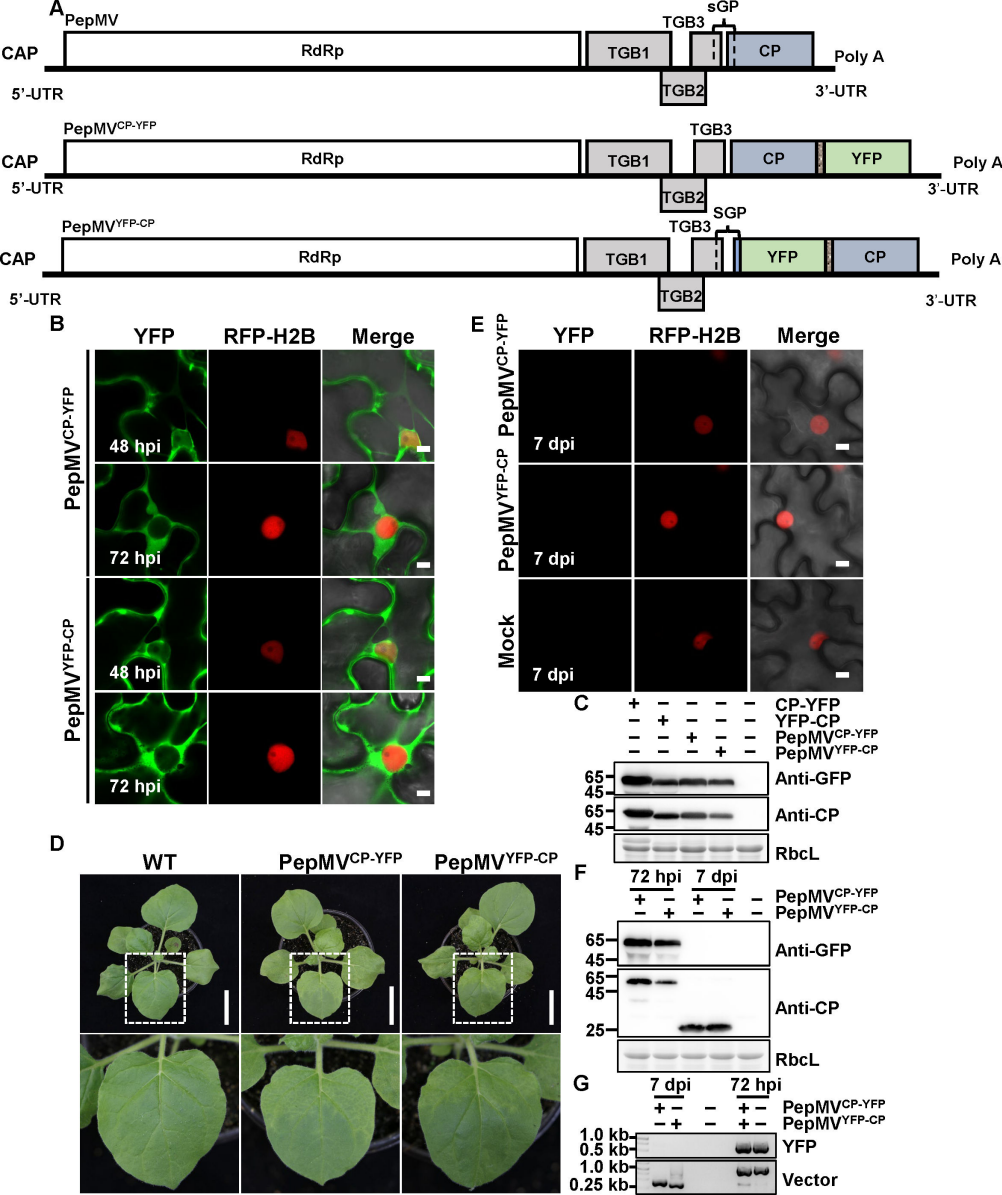
Analysis of PepMV-based vectors expressing CP-fused YFP. (**A**) Schematic representation of the wild-type PepMV genome and the modified vectors PepMV^CP-YFP^ and PepMV^YFP-CP^. The YFP open reading frame was fused to the CP (coat protein) gene via a flexible 3Gly linker in both configurations. (**B**) Subcellular localization of fusion proteins. RFP-H2B transgenic *N. benthamiana* leaves were infiltrated with *A. tumefaciens* suspensions carrying the indicated constructs and examined by confocal laser scanning microscopy at 48 and 72 hpi. Scale bar = 5 µm. (**C**) Immunoblot analysis of fusion protein expression in infiltrated leaf tissues at 72 hpi. (**D**) Representative symptoms of systemically infected leaves at 7 dpi. Scale bar = 6 cm. (**E**) Confocal laser scanning microscopy analysis of systemic leaves at 7 dpi. Scale bar = 5 µm. (**F**) Immunoblot analysis of protein expression in systemically infected leaves at 7 dpi. (**G**) RT-PCR detection of viral RNA and verification of insert integrity. Total RNA from systemic leaves (7 dpi) was analyzed by RT-PCR with primer sets specific for the PepMV vector backbone and the YFP insert. All *A. tumefaciens* suspensions were adjusted to an OD₆₀₀ of 0.5. A volume of 0.5 mL of each suspension was infiltrated per leaf. Experiments were repeated three times independently. For microscopy, more than 20 cells were examined per sample per replicate; representative images are shown.

To investigate the localization dynamics of RdRP and CP during PepMV infection, we generated a series of viral vectors expressing fluorescent fusion proteins. For RdRP, we created both N- and C-terminal YFP fusions (PepMV^YFP-RdRP^ and PepMV^RdRP-YFP^), with the native RdRP genomic promoter driving expression of the YFP-RdRP construct, while the RdRP-YFP fusion incorporated a 3Gly linker between the protein and fluorescent tag. Similarly, for CP, we engineered analogous N- and C-terminal fusion vectors (PepMV^YFP-CP^ and PepMV^CP-YFP^), maintaining the same expression and linker strategies to ensure consistent comparison of fusion orientation effects (Fig. 7A and 8A). The subcellular localization patterns observed using these viral vectors reproduced the overall distributions seen in transient expression assays under PepMV infection (Fig. 2D and E).

For RdRP, PepMV^RdRP-YFP^ produced only weak fluorescent signals in the cytoplasm at 48 and 72 hpi. In contrast, PepMV^YFP-RdRP^ exhibited pronounced nuclear localization, a pattern consistent with that observed upon transient expression of YFP-RdRP (Fig. 7B). Western blot analysis confirmed the expression of both fusion proteins at 72 hpi (Fig. 7C). For CP, viral vectors with N- and C-terminal YFP fusions were similarly fused (Fig. 8A). Compared with transiently expressed YFP-CP (Fig. 1E), which produced large and abundant droplet-like condensates, PepMV^YFP-CP^ showed no detectable puncta at 48 hpi and displayed only a small number of tiny puncta at later stages of infection (Fig. 8B), indicating that transient overexpression exaggerates CP aggregation behavior. All RdRP- and CP-based vectors established systemic infection and induced typical mosaic symptoms (Fig. 7D and 8D). However, systemic leaves lacked detectable fluorescence or full-length fusion proteins (Fig. 7E and F; 8E and F). RT-PCR analysis revealed that the viral RNA initially contained the YFP insert at 72 hpi, but this insert was lost by 7 dpi, yielding only the wild-type RNA sequence (Fig. 7G and 8G).

## DISCUSSION

Understanding the authentic subcellular behavior of viral proteins requires experimental systems that faithfully recapitulate the molecular context of infection. Consistent with prior reports highlighting the limitations of Agrobacterium-mediated transient overexpression (26, 27), our transient assays revealed pronounced discrepancies compared with PepMV infection: RdRP was poorly detectable, TGB2 accumulation declined over time, and hallmark infection-associated features—such as plasmodesmata-associated signals and perinuclear, VRC-like aggregates—were consistently absent (Fig. 1; Fig. S1B and D). To partially approximate infection conditions, YFP-tagged viral proteins were co-expressed with the infectious PepMV clone, which enhanced viral protein accumulation and restored several features, including peripheral puncta of TGB2/TGB3 and plasmodesmal-proximal enrichment of TGB1 (Fig. 2A through C; Fig. S1). However, as expression was driven by a constitutive promoter and not genetically linked to viral replication, spatiotemporal dynamics remained decoupled from the native viral life cycle (15, 28, 29). Thus, co-expression only partially restored localization patterns but did not fully recapitulate authentic infection organization (17, 19, 30–32).

To overcome these limitations, genome-integrated PepMV expression systems under viral subgenomic promoters were employed, following strategies used in related potexviruses (29, 33, 34). We used this infectious tagging platform to observe infection-dependent localization patterns. These patterns were absent or only partially reproduced under transient expression. Under genome-integrated expression, PepMV proteins displayed a coordinated, infection-dependent redistribution pattern characteristic of active replication and movement. At later stages of infection, multiple viral proteins converged into prominent perinuclear assemblies resembling the VRCs or X-bodies described for PVX, reflecting large-scale membrane remodeling coupled to viral replication. At earlier stages, movement-associated proteins preferentially localized to discrete peripheral puncta and plasmodesmata-proximal regions, consistent with their proposed roles in cell-to-cell transport. While fluorescence imaging alone cannot definitively assign these structures, their distribution is consistent with ER-derived motile structures and PD-associated localization reported for TGB proteins of PVX and other potexviruses (35–37), suggesting PepMV employs conserved movement strategies dependent on active infection, genome replication, and host-driven membrane remodeling.

This genome-integrated system provides two key advantages. First, fusion proteins are expressed at near-physiological levels, reducing mislocalization and non-specific aggregation seen with strong constitutive promoters (38–42). Second, expression is coupled to viral replication and host membrane remodeling, ensuring physiologically relevant virus–host interactions. Notably, CP condensation differed between transient and genome-integrated expression. Transient overexpression of CP-YFP produced large droplet-like aggregates, whereas PepMV^YFP-CP^ showed no detectable puncta at 48 hpi and only a limited number of small puncta at later stages of infection. (Fig. 2E and 8B). This aligns with prior studies showing that excessive protein abundance can artificially induce LLPS-like condensates (43, 44), indicating that large droplets under transient expression are non-physiological artifacts.

A limitation of the PepMV infectious tagging system is the instability of reporter inserts during systemic infection. While plants developed systemic symptoms and viral RNA was detected in upper leaves, YFP signals and fusion proteins were absent, with PCR confirming precise deletion of the reporter cassette. This phenomenon is consistent with potexvirus vectors frequently losing foreign inserts during systemic movement (45, 46). Beyond recombination, host RNA surveillance pathways, including nonsense-mediated decay, may recognize structurally aberrant or elongated viral RNAs, contributing to selective removal (47–50). These insights inform strategies for improving insert stability and highlight selective pressures on PepMV genome maintenance.

A major advancement is the PepMV genome-integrated fluorescent tagging system, PepMV^B^, which maintains stable fluorescence during systemic infection. Recombinant viruses carrying TGB2 C-terminal fusions (PepMV^TGB2-sGFP^) preserved infectivity and fluorescence, enabling the first visualization of TGB2 subcellular localization during natural infection. In contrast, N-terminal fusions disrupted membrane insertion and topology, preventing systemic infection, consistent with the U-shaped TGB2 topology in which both N- and C-termini face the cytoplasm (51, 52). As proof of concept, a dual-fluorescence vector co-expressing PepMV^YFP-TGB1-CP-mCherry^ allows simultaneous visualization of multiple viral proteins (Fig. S2). This system could be adapted for future applications, such as BiFC or FRET, providing a framework for quantitative and dynamic analysis of protein interactions under authentic infection conditions.

This study makes three key contributions to plant virology. First, we developed a robust, replicating PepMV-based vector platform, notably PepMV^B^, overcoming the limitations of transient expression. Second, we provide novel insights into PepMV protein behavior under near-physiological conditions, correcting misconceptions from overexpression artifacts. Third, we reveal the spatial division of labor among viral movement proteins in assembling functional replication complexes. Collectively, this work advances understanding of PepMV pathogenesis and delivers a versatile methodological toolkit applicable to other recalcitrant viral systems, laying the groundwork for innovative antiviral strategies (53–56).

## MATERIALS AND METHODS

### Plant materials and growth conditions

Wild-type and transgenic *N. benthamiana* seedlings were grown in a climate chamber with 60% relative humidity under a 15 h light/9 h dark photoperiod as described previously (57). The transgenic RFP-H2B line was a gift from Michael M. Goodin (University of Kentucky, USA).

### Plasmid constructs

#### Transient expression vectors

The coding sequences of PepMV (accession KY031324) RdRP, TGB1, TGB2, TGB3, and CP were amplified by PCR from a previously constructed infectious clone using Phanta DNA Polymerase (Vazyme, China) and gene-specific primers (14, 58, 59) (Table S1). For Gateway cloning, the purified PCR products were first recombined into the pDONR221 entry vector using BP Clonase II (Invitrogen). The inserts in pDONR221 were then transferred into the destination vectors: all constructs (RdRP, TGB1, TGB2, TGB3, and CP) were transferred into pEarleyGate-101 (for C-terminal YFP fusions), while RdRP and TGB1 were additionally transferred into pEarleyGate-104 (for N-terminal YFP fusions) using LR Clonase II (Invitrogen). For In-Fusion cloning (Takara Bio), the amplified fragments of YFP-TGB2, YFP-TGB3, or YFP-CP were inserted into the pCAMBIA1300 vector linearized at the *Kpn* I and *Pst* I sites.

#### PepMV-based viral expression vectors

Recombinant PepMV vectors were constructed using overlapping extension PCR and in-fusion cloning. For N-terminal fusions, the YFP fragment was fused to a 45-nt sequence from the N-terminus of RdRP and cloned into the *Sca* I site (PepMV^YFP-RdRP^), or to the TGB1 subgenomic promoter (PepMV^SGP192^) and cloned into the *Sma* I site (PepMV^YFP-TGB1^). For C-terminal fusions, the YFP fragment was fused to the 108-nt C-terminal fragment of RdRP at the *Sca* I site (PepMV^RdRP-YFP^), to the 30-nt C-terminal fragment of TGB3 at the *Sca* I site (PepMV^TGB3-YFP^), or to the 36-nt C-terminal fragment of CP at the *Apa* I site (PepMV^CP-YFP^). Additionally, the CP subgenomic promoter (PepMV^SP1^) was fused to YFP at the *Apa* I site (PepMV^YFP-CP^). For TGB2 tagging, the GFP coding sequence (amplified from tobacco mosaic virus [TMV]-GFP vector) was inserted into the PepMV vector at the *Xho* I, *Apa* I, and *BamH* I sites, generating PepMV^A^, PepMV^B^, and PepMV^C^, respectively. The TGB1 subgenomic promoter (PepMV^SGP192^), the TGB2 fragment, and the GFP fragment were then assembled via overlapping PCR and cloned into these sites to generate PepMV^sGFP-TGB2^ and PepMV^TGB2-sGFP^.

### *Agrobacterium* infiltration and inoculation

Viral infections were performed by agroinfiltration of PepMV infectious clones into 4-week-old plants as described (25). The infiltration buffer contained 10 mM MgCl₂, 10 mM MES (pH 5.6), and 150 μM acetosyringone in sterile water. *A. tumefaciens* strains harboring the respective plasmids were cultured overnight at 28°C in appropriate antibiotic media. Bacterial cultures were centrifuged and resuspended in infiltration buffer to an optical density at 600 nm (OD_600_) of 0.5 for all constructs, except for RdRP-related constructs, which were adjusted to OD_600_ = 1.0. The suspensions were incubated for 2–3 h at room temperature before infiltration. For PepMV^A^, PepMV^B^, PepMV^C^, PepMV^sGFP-TGB2^, and PepMV^TGB2-sGFP^, the corresponding *A. tumefaciens* strains were co-infiltrated into *N. benthamiana* leaves. Agroinfiltrated leaves were visualized under a long-wavelength UV lamp (UVP) at 2 dpi and photographed through a yellow filter.

### Confocal laser scanning microscopy

The observation of subcellular localization of viral proteins was conducted by confocal laser scanning microscopy as described previously (60). The genes of interest were transiently expressed in the transgenic RFP-H2B *N. benthamiana* leaves, and 1–2 cm^2^ sections of the infiltrated leaves were excised and examined by confocal laser scanning microscopy (Carl Zeiss LSM 980, Germany) at 48 and 72 hpi. YFP was excited at 518 nm, and the emitted light was captured at 565–585 nm. RFP was excited at 552 nm, and the emitted light was captured at 590–630 nm. At least 20 cells were examined for each experiment. The collected images were analyzed using the ZEN 2 (Carl Zeiss Microscope GmbH- 2011) software.

### Western blot analysis

Total protein was extracted from infiltrated leaf tissues using a previously described method (60). Proteins were separated by 12% sodium dodecyl sulfate-polyacrylamide gel electrophoresis and transferred onto nitrocellulose membranes. The membranes were immunoblotted with the following primary antibodies: mouse anti-GFP (1:5,000, Roche, catalog no. 11814460001), mouse anti-mCherry (1:5,000, Abcam, catalog no. ab167453), and custom-made mouse anti-PepMV CP polyclonal antibody (1:5,000; [25]). Subsequently, the membranes were incubated with horseradish peroxidase-conjugated anti-mouse IgG secondary antibody (1:5,000, Easybio, catalog no. BE0102). Protein signals were detected using an enhanced chemiluminescence substrate.

### RNA extraction and qRT-PCR

Total RNA extraction was performed as described previously (60). RT-PCR and qRT-PCR were performed according to previously described methods (47, 61). All primers used for qRT-PCR are listed in Table S1.

## Data Availability

The authors confirm that the data supporting the findings of this study are available and included within this article.

## SUPPLEMENTAL MATERIAL

Supplemental material jvi.01694-25-s0001.docx Fig. S1 and S2; Table S1.

ASM does not own the copyrights to Supplemental Material that may be linked to, or accessed through, an article. The authors have granted ASM a non-exclusive, world-wide license to publish the Supplemental Material files. Please contact the corresponding author directly for reuse.

## References

[1] Jones RAC, Koenig R, Lesemann DE. 1980. Pepino mosaic virus, a new potexvirus from pepino ( Solanum muricatum ). Annals of Applied Biology 94:61–68. doi:10.1111/j.1744-7348.1980.tb03896.x

[2] Blystad D-R, van der Vlugt R, Alfaro-Fernández A, del Carmen Córdoba M, Bese G, Hristova D, Pospieszny H, Mehle N, Ravnikar M, Tomassoli L, Varveri C, Nielsen SL. 2015. Host range and symptomatology of pepino mosaic virus strains occurring in Europe. Eur J Plant Pathol 143:43–56. doi:10.1007/s10658-015-0664-1

[3] Hanssen IM, Thomma B. 2010. Pepino mosaic virus: a successful pathogen that rapidly evolved from emerging to endemic in tomato crops. Mol Plant Pathol 11:179–189. doi:10.1111/j.1364-3703.2009.00600.x 20447268 PMC6640333

[4] van der Vlugt RAA, Stijger CCMM, Verhoeven JTJ, Lesemann D-E. 2000. First report of pepino mosaic virus on tomato. Plant Dis 84:103. doi:10.1094/PDIS.2000.84.1.103C 30841211

[5] Klap C, Luria N, Smith E, Hadad L, Bakelman E, Sela N, Belausov E, Lachman O, Leibman D, Dombrovsky A. 2020. Tomato brown rugose fruit virus contributes to enhanced pepino mosaic virus titers in tomato plants. Viruses 12:879. doi:10.3390/v12080879 32796777 PMC7472245

[6] Elvira González L, Peiró R, Rubio L, Galipienso L. 2021. Persistent southern tomato virus (STV) interacts with cucumber mosaic and/or pepino mosaic virus in mixed- infections modifying plant symptoms, viral titer and small RNA accumulation. Microorganisms 9:689. doi:10.3390/microorganisms9040689 33810543 PMC8066132

[7] Aguilar JM, Hernández-Gallardo MD, Cenis JL, Lacasa A, Aranda MA. 2002. Complete sequence of the pepino mosaic virus RNA genome. Arch Virol 147:2009–2015. doi:10.1007/s00705-002-0848-9 12376761

[8] Osman TAM, Olsthoorn RCL, Livieratos IC. 2012. In vitro template-dependent synthesis of pepino mosaic virus positive- and negative-strand RNA by its RNA-dependent RNA polymerase. Virus Res 167:267–272. doi:10.1016/j.virusres.2012.05.008 22617023

[9] Sempere RN, Gómez-Aix C, Ruíz-Ramón F, Gómez P, Hasiów-Jaroszewska B, Sánchez-Pina MA, Aranda MA. 2016. Pepino mosaic virus RNA-dependent RNA polymerase POL domain is a hypersensitive response-like elicitor shared by necrotic and mild isolates. Phytopathology 106:395–406. doi:10.1094/PHYTO-10-15-0277-R 26667188

[10] Morozov SYu, Solovyev AG. 2003. Triple gene block: modular design of a multifunctional machine for plant virus movement. J Gen Virol 84:1351–1366. doi:10.1099/vir.0.18922-0 12771402

[11] Mathioudakis Matthaios M, Khechmar S, Owen CA, Medina V, Ben Mansour K, Tomaszewska W, Spanos T, Sarris PF, Livieratos IC. 2018. A thioredoxin domain-containing protein interacts with Pepino mosaic virus triple gene block protein 1. Int J Mol Sci 19:3747. doi:10.3390/ijms19123747 30477269 PMC6320799

[12] Hasiów-Jaroszewska B, Czerwoniec A, Pospieszny H, Elena SF. 2011. Tridimensional model structure and patterns of molecular evolution of pepino mosaic virus TGBp3 protein. Virol J 8:318. doi:10.1186/1743-422X-8-318 21702943 PMC3132167

[13] Agirrezabala X, Méndez-López E, Lasso G, Sánchez-Pina MA, Aranda M, Valle M. 2015. The near-atomic cryoEM structure of a flexible filamentous plant virus shows homology of its coat protein with nucleoproteins of animal viruses. eLife 4:e11795. doi:10.7554/eLife.11795 26673077 PMC4739775

[14] Sempere RN, Gómez P, Truniger V, Aranda MA. 2011. Development of expression vectors based on pepino mosaic virus. Plant Methods 7:6. doi:10.1186/1746-4811-7-6 21396092 PMC3065447

[15] Mathioudakis M.M, Rodríguez-Moreno L, Sempere RN, Aranda MA, Livieratos I. 2014. Multifaceted capsid proteins: multiple interactions suggest multiple roles for pepino mosaic virus capsid protein. Mol Plant Microbe Interact 27:1356–1369. doi:10.1094/MPMI-07-14-0195-R 25162316

[16] Méndez-López E, Donaire L, Gosálvez B, Díaz-Vivancos P, Sánchez-Pina MA, Tilsner J, Aranda MA. 2023. Tomato SlGSTU38 interacts with the PepMV coat protein and promotes viral infection. New Phytol 238:332–348. doi:10.1111/nph.18728 36631978

[17] Tilsner Jens, Linnik O, Wright KM, Bell K, Roberts AG, Lacomme C, Santa Cruz S, Oparka KJ. 2012. The TGB1 movement protein of Potato virus X reorganizes actin and endomembranes into the X-body, a viral replication factory. Plant Physiol 158:1359–1370. doi:10.1104/pp.111.189605 22253256 PMC3291258

[18] Verchot J. 2022. Potato virus X: A global potato-infecting virus and type member of the potexvirus genus. Mol Plant Pathol 23:315–320. doi:10.1111/mpp.13163 34791766 PMC8828454

[19] Tilsner J, Linnik O, Louveaux M, Roberts IM, Chapman SN, Oparka KJ. 2013. Replication and trafficking of a plant virus are coupled at the entrances of plasmodesmata. J Cell Biol 201:981–995. doi:10.1083/jcb.201304003 23798728 PMC3691464

[20] Wu X, Liu J, Chai M, Wang J, Li D, Wang A, editor. 2019. The potato virus X TGBp2 protein plays dual functional roles in viral replication and movement. J Virol 93:e01635–18. doi:10.1128/JVI.01635-18 30541845 PMC6384063

[21] Li F, Sun Z, Zhou X. 2024. Editorial: recent advances in plant virology. Virology (Auckl) 595:110099. doi:10.1016/j.virol.2024.110099 38701717

[22] Wu J, Zhang Y, Li F, Zhang X, Ye J, Wei T, Li Z, Tao X, Cui F, Wang X, Zhang L, Yan F, Li S, Liu Y, Li D, Zhou X, Li Y. 2024. Plant virology in the 21st century in China: recent advances and future directions. JIPB 66:579–622. doi:10.1111/jipb.13580 37924266

[23] Wang Y, Mei Y, Li F. 2025. Engineering nucleotide‐binding leucine‐rich repeat immune receptors to achieve broad‐spectrum disease resistance in plants. New Plant Protection. doi:10.1002/npp2.70023

[24] Martin K, Kopperud K, Chakrabarty R, Banerjee R, Brooks R, Goodin MM. 2009. Transient expression in nicotiana benthamiana fluorescent marker lines provides enhanced definition of protein localization, movement and interactions in planta. Plant J 59:150–162. doi:10.1111/j.1365-313X.2009.03850.x 19309457

[25] He H, Ge L, Li Z, Zhou X, Li F. 2023. Pepino mosaic virus antagonizes plant m6A modification by promoting the autophagic degradation of the m6A writer HAKAI. aBIOTECH 4:83–96. doi:10.1007/s42994-023-00097-6 37581026 PMC10423194

[26] Müller L, Hoppe T. 2024. UPS-dependent strategies of protein quality control degradation. Trends Biochem Sci 49:859–874. doi:10.1016/j.tibs.2024.06.006 38945729

[27] Vega MC, ed. 2016. Advanced technologies for protein complex production and characterization. Adv Exp Med Biol Available from. doi:10.1007/978-3-319-27216-0 27532079

[28] Mathioudakis Matthaios M, Veiga RSL, Canto T, Medina V, Mossialos D, Makris AM, Livieratos I. 2013. Pepino mosaic virus triple gene block protein 1 (TGBp1) interacts with and increases tomato catalase 1 activity to enhance virus accumulation. Mol Plant Pathol 14:589–601. doi:10.1111/mpp.12034 23634807 PMC6638622

[29] Ruiz-Ramón F, Sempere RN, Méndez-López E, Sánchez-Pina MA, Aranda MA. 2019. Second generation of pepino mosaic virus vectors: improved stability in tomato and a wide range of reporter genes. Plant Methods 15:58. doi:10.1186/s13007-019-0446-4 31149024 PMC6537163

[30] Crawford KM, Zambryski PC. 2001. Non-targeted and targeted protein movement through plasmodesmata in leaves in different developmental and physiological states. Plant Physiol 125:1802–1812. doi:10.1104/pp.125.4.1802 11299360 PMC88836

[31] Oparka KJ, Roberts AG, Boevink P, Santa Cruz S, Roberts I, Pradel KS, Imlau A, Kotlizky G, Sauer N, Epel B. 1999. Simple, but not branched, plasmodesmata allow the nonspecific trafficking of proteins in developing tobacco leaves. Cell 97:743–754. doi:10.1016/s0092-8674(00)80786-2 10380926

[32] Samuels TD, Ju HJ, Ye CM, Motes CM, Blancaflor EB, Verchot-Lubicz J. 2007. Subcellular targeting and interactions among the potato virus X TGB proteins. Virology (Auckl) 367:375–389. doi:10.1016/j.virol.2007.05.022 17610926

[33] Abrahamian P, Hammond J, Hammond RW. 2021. Development and optimization of a pepino mosaic virus-based vector for rapid expression of heterologous proteins in plants. Appl Microbiol Biotechnol 105:627–645. doi:10.1007/s00253-020-11066-0 33394156

[34] Chen X, Zaro JL, Shen WC. 2013. Fusion protein linkers: property, design and functionality. Adv Drug Deliv Rev 65:1357–1369. doi:10.1016/j.addr.2012.09.039 23026637 PMC3726540

[35] Howard AR, Heppler ML, Ju HJ, Krishnamurthy K, Payton ME, Verchot-Lubicz J. 2004. Potato virus X TGBp1 induces plasmodesmata gating and moves between cells in several host species whereas CP moves only in N. benthamiana leaves. Virology (Auckl) 328:185–197. doi:10.1016/j.virol.2004.06.039 15464839

[36] Ju H-J, Samuels TD, Wang Y-S, Blancaflor E, Payton M, Mitra R, Krishnamurthy K, Nelson RS, Verchot-Lubicz J. 2005. The potato virus X TGBp2 movement protein associates with endoplasmic reticulum-derived vesicles during virus infection. Plant Physiol 138:1877–1895. doi:10.1104/pp.105.066019 16055678 PMC1183379

[37] Schepetilnikov MV, Manske U, Solovyev AG, Zamyatnin AA, Schiemann J, Morozov SY. 2005. The hydrophobic segment of potato virus X TGBp3 is a major determinant of the protein intracellular trafficking. J Gen Virol 86:2379–2391. doi:10.1099/vir.0.80865-0 16033986

[38] Alsheikhly AR, Andersson T, Perlmann P. 1985. Virus-dependent cellular cytotoxicity in vitro. Mechanisms of induction and effector cell characterization. Scand J Immunol 21:329–335. doi:10.1111/j.1365-3083.1985.tb01438.x 3873684

[39] Egelkrout E, Rajan V, Howard JA. 2012. Overproduction of recombinant proteins in plants. Plant Sci 184:83–101. doi:10.1016/j.plantsci.2011.12.005 22284713

[40] Gong Q, Wang Y, Jin Z, Hong Y, Liu Y. 2022. Transcriptional and post-transcriptional regulation of RNAi-related gene expression during plant-virus interactions. Stress Biol 2:33. doi:10.1007/s44154-022-00057-y 37676459 PMC10441928

[41] Pesti R, Kontra L, Paul K, Vass I, Csorba T, Havelda Z, Várallyay É. 2019. Differential gene expression and physiological changes during acute or persistent plant virus interactions may contribute to viral symptom differences. PLoS One 14:e0216618. doi:10.1371/journal.pone.0216618 31051010 PMC6499435

[42] Ray S, Casteel CL. 2022. Effector-mediated plant-virus-vector interactions. Plant Cell 34:1514–1531. doi:10.1093/plcell/koac058 35277714 PMC9048964

[43] Alberti S, Hyman AA. 2021. Biomolecular condensates at the nexus of cellular stress, protein aggregation disease and ageing. Nat Rev Mol Cell Biol 22:196–213. doi:10.1038/s41580-020-00326-6 33510441

[44] Shin Y, Brangwynne CP. 2017. Liquid phase condensation in cell physiology and disease. Science 357:eaaf4382. doi:10.1126/science.aaf4382 28935776

[45] Bedoya LC, Daròs JA. 2010. Stability of tobacco etch virus infectious clones in plasmid vectors. Virus Res 149:234–240. doi:10.1016/j.virusres.2010.02.004 20152868

[46] Boevink P, Santa Cruz S, Hawes C, Harris N, Oparka KJ. 1996. Virus‐mediated delivery of the green fluorescent protein to the endoplasmic reticulum of plant cells. Plant J 10:935–941. doi:10.1046/j.1365-313X.1996.10050935.x

[47] Chen Y, Jia M, Ge L, Li Z, He H, Zhou X, Li F. 2024. A negative feedback loop compromises NMD‐mediated virus restriction by the autophagy pathway in plants. Adv Sci (Weinh) 11:2400978. doi:10.1002/advs.202400978 39189522 PMC11348178

[48] Garcia D, Garcia S, Voinnet O. 2014. Nonsense-mediated decay serves as a general viral restriction mechanism in plants. Cell Host & Microbe 16:391–402. doi:10.1016/j.chom.2014.08.001 25155460 PMC7185767

[49] Ge L, Zhou X, Li F. 2024. Plant-virus arms race beyond RNA interference. Trends Plant Sci 29:16–19. doi:10.1016/j.tplants.2023.10.014 37953079

[50] May JP, Simon AE. 2021. Targeting of viral RNAs by Upf1-mediated RNA decay pathways. Curr Opin Virol 47:1–8. doi:10.1016/j.coviro.2020.11.002 33341474 PMC8744489

[51] Hsu H-T, Chou Y-L, Tseng Y-H, Lin Y-H, Lin T-M, Lin N-S, Hsu Y-H, Chang B-Y. 2008. Topological properties of the triple gene block protein 2 of Bamboo mosaic virus. Virology (Auckl) 379:1–9. doi:10.1016/j.virol.2008.06.019 18639913

[52] Zamyatnin AA Jr, Solovyev AG, Bozhkov PV, Valkonen JPT, Morozov SYu, Savenkov EI. 2006. Assessment of the integral membrane protein topology in living cells. Plant J 46:145–154. doi:10.1111/j.1365-313X.2006.02674.x 16553902

[53] Citovsky V, Lee L-Y, Vyas S, Glick E, Chen M-H, Vainstein A, Gafni Y, Gelvin SB, Tzfira T. 2006. Subcellular localization of interacting proteins by bimolecular fluorescence complementation in planta. J Mol Biol 362:1120–1131. doi:10.1016/j.jmb.2006.08.017 16949607

[54] Hasiów-Jaroszewska B, Borodynko N, Jackowiak P, Figlerowicz M, Pospieszny H. 2011. Single mutation converts mild pathotype of the Pepino mosaic virus into necrotic one. Virus Res 159:57–61. doi:10.1016/j.virusres.2011.04.008 21536084

[55] Jares-Erijman EA, Jovin TM. 2003. FRET imaging. Nat Biotechnol 21:1387–1395. doi:10.1038/nbt896 14595367

[56] Kerppola TK. 2008. Bimolecular fluorescence complementation (BiFC) analysis as a probe of protein interactions in living cells. Annu Rev Biophys 37:465–487. doi:10.1146/annurev.biophys.37.032807.125842 18573091 PMC2829326

[57] Li Z, Yang X, Li W, Wen Z, Duan J, Jiang Z, Zhang D, Xie X, Wang X, Li F, Li D, Zhang Y. 2022. SAMDC3 enhances resistance to Barley stripe mosaic virus by promoting the ubiquitination and proteasomal degradation of viral γb protein. New Phytol 234:618–633. doi:10.1111/nph.17993 35075654

[58] Hasiów-Jaroszewska B, Borodynko N, Pospieszny H. 2009. Infectious RNA transcripts derived from cloned cDNA of a pepino mosaic virus isolate. Arch Virol 154:853–856. doi:10.1007/s00705-009-0368-y 19333548

[59] Hasiów-Jaroszewska B, Borodynko N. 2012. Characterization of the necrosis determinant of the European genotype of pepino mosaic virus by site-specific mutagenesis of an infectious cDNA clone. Arch Virol 157:337–341. doi:10.1007/s00705-011-1162-1 22068882

[60] Li F, Zhang C, Li Y, Wu G, Hou X, Zhou X, Wang A. 2018. Beclin1 restricts RNA virus infection in plants through suppression and degradation of the viral polymerase. Nat Commun 9:1268. doi:10.1038/s41467-018-03658-2 29593293 PMC5871769

[61] Gong P, Tan H, Zhao S, Li H, Liu H, Ma Y, Zhang X, Rong J, Fu X, Lozano-Durán R, Li F, Zhou X. 2021. Geminiviruses encode additional small proteins with specific subcellular localizations and virulence function. Nat Commun 12:4278. doi:10.1038/s41467-021-24617-4 34257307 PMC8277811

