# Supplementary material for: Viral vector-based imaging uncovers infection-induced re-localization of pepino mosaic virus proteins
Source: J Virol. 2026 Mar 24;100(4):e01694-25. doi: 10.1128/jvi.01694-25 (PMC13098275; doi:10.1128/jvi.01694-25)
Supplement: Supplemental material — Fig. S1 and S2; Table S1. [file jvi.01694-25-s0001.docx]

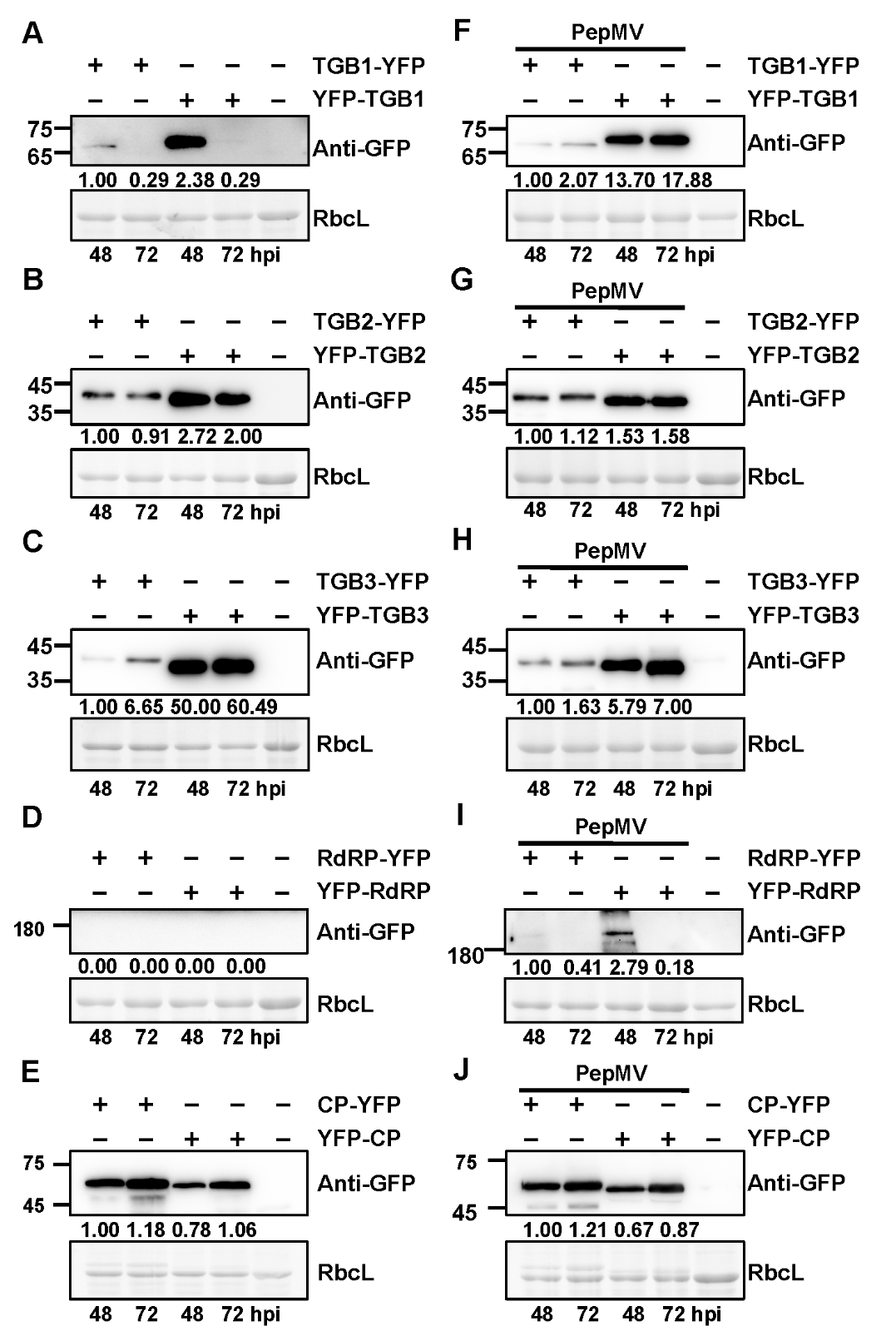


**FIG S1** Dynamic accumulation of PepMV viral fusion proteins in the presence or absence of viral infection. Wild-type *N. benthamiana* leaves were infiltrated with *A. tumefaciens* suspensions to co-express the following YFP-fusion proteins: RdRP-YFP and YFP-RdRP **(A, F)**, TGB1-YFP and YFP-TGB1 **(B, G)**, TGB2-YFP and YFP-TGB2 **(C, H)**, TGB3-YFP and YFP-TGB3 **(D, I)**, or CP-YFP and YFP-CP **(E, J)**, together with either an empty vector PGR (Mock; a–e) or a PepMV infectious clone **(F–J)**. Total protein was extracted from infiltrated leaf tissues at 48 and 72 hpi and analyzed by immunoblotting using an anti-GFP antibody. Loaded sample volumes were 20 µl **(A, F)** or 10 µl (all other panels). The Rubisco large subunit, visualized by UV imaging, served as the loading control. Protein levels were quantified using ImageJ and normalized to the corresponding mock condition set as 1.00. All immunoblotting experiments were repeated at least three times with biological replicates derived from three independent infiltrations. One representative blot is shown.


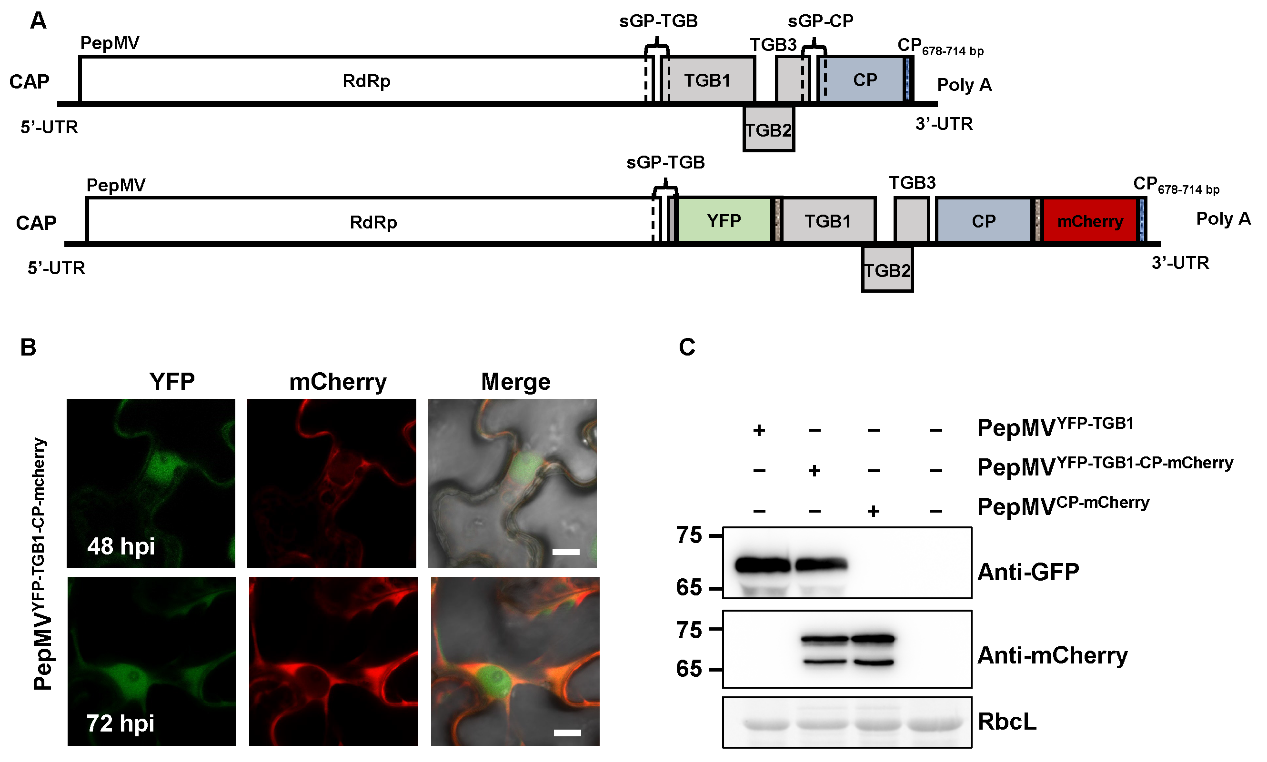


**FIG S2** Bimolecular labeling and co-expression analysis of the PepMV movement protein TGB1 and coat protein (CP) fusions. **(A)** Schematic representation of the wild-type PepMV genome and the modified vector PepMV^TGB1-YFP-CP-mCherry^. The YFP open reading frame was fused to the C-terminus of the *TGB1* gene via a 3Gly linker peptide. The mCherry open reading frame was fused to the C-terminus of the *CP* gene via a separate 3×Gly linker peptide. **(B)** Subcellular localization and interaction analysis. RFP-H2B transgenic *N.benthamiana* leaves were co-infiltrated with *A. tumefaciens* suspensions carrying the indicated constructs and examined by Confocal laser scanning microscopy at 48 and 72 hpi. Scale bar = 5 µm. **(C)** Immunoblot analysis of dual fusion protein expression in infiltrated leaf tissues at 72 hpi. Membranes were probed with both anti-GFP and anti-RFP antibodies to detect YFP and mCherry fusion proteins, respectively. All *A. tumefaciens* suspensions were adjusted to an OD₆₀₀ of 0.5. A volume of 0.5 mL of each bacterial suspension was infiltrated per leaf. Experiments were repeated three times independently. For microscopy, more than 20 cells were examined per sample per replicate; representative images are shown.

**Table S1. Primers used for qualitative RT-PCR and vector construction in this study.**

| Primer Name | Sequence (5' → 3') | Purpose |
| --- | --- | --- |
| YFP-TGB1-InFusion-F | TGCTTTCAAAATGCAACATG | Construction of YFP-TGB1 fusion |
| YFP-TGB1-InFusion-R | ACTTGTGAAATTTTATTAGC | Construction of YFP-TGB1 fusion |
| TGB3-YFP-InFusion-F | TCATTCTTACATTGGGTATC | Construction of TGB3-YFP fusion |
| TGB3-YFP-InFusion-F | GTCCAGTTTCATTAGCAGCC | Construction of TGB3-YFP fusion |
| CP- YFP- F | TCATTCTTACATTGGGTATC | Construction of CP-YFP fusion |
| CP-YFP-R | AGGCGATTAAGTTGGGTAAC | Construction of CP-YFP fusion |
| YFP-CP-F | TCATTCTTACATTGGGTATC | Construction of YFP-CP- fusion |
| YFP-CP -R | AGGCGATTAAGTTGGGTAAC | Construction of YFP-CP- fusion |
| YFP-Insert-F | ATGGTGAGCAAGGGCGAG | RT-PCR for detecting YFP insert in viral RNA |
| YFP-Insert-R | CTTGTACAGCTCGTCCATG | RT-PCR for detecting YFP insert in viral RNA |
